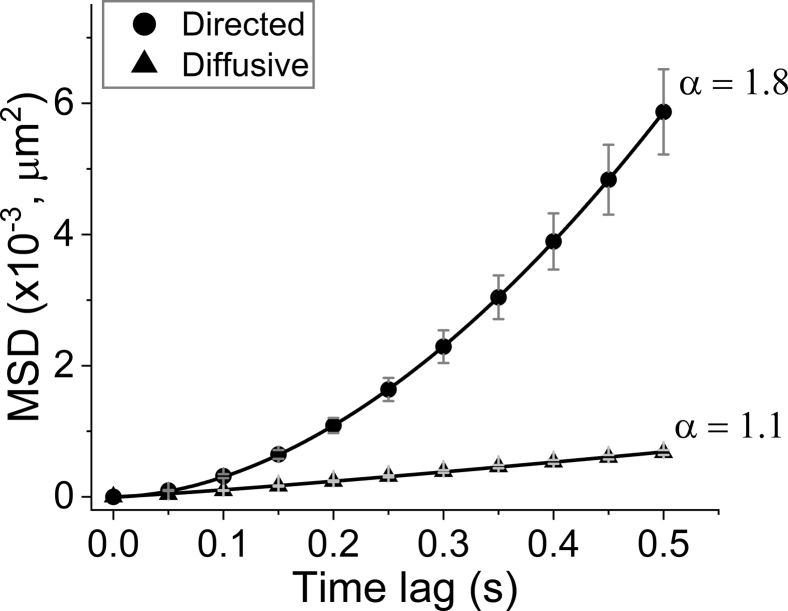# The sodium proton exchanger NHE9 regulates phagosome maturation and bactericidal activity in macrophages

**DOI:** 10.1016/j.jbc.2022.102150

**Published:** 2022-06-16

**Authors:** Habiba S. Shamroukh, Nabrah Lone, Muaaz Akhtar, Alhareth Altayib, Shelby Sutliff, Zahraa Kassem, Suvranta K. Tripathy, Kalyan C. Kondapalli

**Affiliations:** Department of Natural Sciences, University of Michigan-Dearborn, Dearborn, Michigan, USA

**Keywords:** sodium-proton exchange, macrophage, NHE9, SLC9A9, pH, phagosome transport, molecular motor, BMDM, bone marrow–derived macrophage, Ct, cycle threshold, DMEM, Dulbecco’s modified Eagle’s medium, LPS, lipopolysaccharide, MR, Magic Red, MSD, mean-square displacement, TLR, toll-like receptor

## Abstract

Acidification of phagosomes is essential for the bactericidal activity of macrophages. Targeting machinery that regulates pH within the phagosomes is a prominent strategy employed by various pathogens that have emerged as major threats to public health. Nascent phagosomes acquire the machinery for pH regulation through a graded maturation process involving fusion with endolysosomes. Meticulous coordination between proton pumping and leakage mechanisms is crucial for maintaining optimal pH within the phagosome. However, relative to mechanisms involved in acidifying the phagosome lumen, little is known about proton leakage pathways in this organelle. Sodium proton transporter NHE9 is a known proton leakage pathway located on the endosomes. As phagosomes acquire proteins through fusions with endosomes during maturation, NHE9 seemed a promising candidate for regulating proton fluxes on the phagosome. Here, using genetic and biophysical approaches, we show NHE9 is an important proton leakage pathway associated with the maturing phagosome. NHE9 is highly expressed in immune cells, specifically macrophages; however, NHE9 expression is strongly downregulated upon bacterial infection. We show that compensatory ectopic NHE9 expression hinders the directed motion of phagosomes along microtubules and promotes early detachment from the microtubule tracks. As a result, these phagosomes have shorter run lengths and are not successful in reaching the lysosome. In accordance with this observation, we demonstrate that NHE9 expression levels negatively correlate with bacterial survival. Together, our findings show that NHE9 regulates lumenal pH to affect phagosome maturation, and consequently, microbicidal activity in macrophages.

Dynamics of the interactions between bacterial pathogens and the host innate immune system are crucial for determining the trajectory of the infection (1). Macrophages, along with neutrophils and dendritic cells, are key determinants of the innate immune response (2). Macrophage response is characterized by recognizing the infectious agent through pathogen-associated molecular patterns, proliferation of macrophages into the infected tissue followed by internalizing the bacterial pathogen for degradation, and antigen presentation to T-cells (3, 4, 5). The internalized bacteria are entrapped within a vacuole in the macrophage known as the phagosome. The nascent phagosome, a vesicle with lumenal contents resembling the extracellular milieu ensnared by a piece of the macrophage cell membrane, is incompetent to kill the ingested bacteria (6). The phagosome matures through sequential fusions with endosomes resulting in increasing lumenal acidity and changes in phagosome composition while being transported toward the perinuclear region by microtubule-based molecular motor proteins (7, 8). While many bacteria are effectively eliminated within the mature phagosome, several species can survive and adapt (1). A common target of several unrelated bacteria within the phagosome is the machinery that acidifies the phagosome during maturation (1, 9, 10). While the ability of the phagosomes to acidify the lumen has been recognized for more than a century, the determinants of phagosomal pH are not completely known (1, 11). Moreover, in the context of various disease-causing bacteria resisting elimination by targeting phagosomal acidification, identifying and characterizing the regulators of phagosomal pH becomes extremely important.

Shared patterns of molecular modulations in response to a broad range of bacteria offer crucial insight into macrophage defense mechanisms (12). Previous studies in macrophages have shown that expression of the endosomal pH regulator, NHE9, is modulated in response to a variety of bacteria (12, 13, 14, 15, 16, 17). However, functional link(s) between NHE9 expression and macrophage activity have not been examined. NHE9, encoded by the gene SLC9A9, belongs to an ancient family of evolutionarily conserved transport proteins that regulate cellular and organellar pH (18). Currently, 13 distinct mammalian orthologs of sodium proton exchangers (NHEs) have been recognized, each differing in transport kinetics, cellular localization, tissue distribution, and substrate preferences (18, 19, 20, 21). NHE isoform 9 (NHE9), the oldest in the order of evolution of intracellular NHEs, couples the electroneutral transfer of protons out of the endosome lumen to the counter-transport of Na^+^ or K^+^ ions, thereby alkalizing the endosome (18, 22). NHE9’s C-terminal cytoplasmic tail modulates various important cellular signaling pathways *via* its protein interaction network (23, 24, 25). Though roles in vesicular trafficking, synaptic membrane turnover, and modulation of signaling axes have been recognized for NHE9, its functional profile remains incomplete (25, 26, 27, 28). Considering these important roles in cellular physiology, it is not surprising that dysfunction of NHE9 is implicated in various diseases. While loss of NHE9 function is associated with familial autism and attention deficit hyperactivity disorder, gain of NHE9 function underlies subsets of cancers such as glioblastoma and esophageal squamous cell carcinoma (23, 27, 29, 30, 31, 32, 33).

The factors that determine the phagosomal pH remain poorly understood. The acidity within the phagosome is generated by the V-type H^+^-ATPase, which transports 3H^+^ into the lumen by hydrolyzing one ATP molecule (1). However, without counter-ion fluxes, the electric potential generated by the influx of protons would oppose any significant changes in lumenal pH (1). As a result of the sequential fusions with endosomal membranes, it is reasonable to hypothesize that H^+^ leakage on the phagosome is regulated similarly to the endolysosomal system. In this study, we analyzed the functional link between NHE9 and bactericidal activity of macrophages and found that classical activation of macrophages resulted in pronounced downregulation of NHE9 in macrophages. We also examined the importance of NHE9 downregulation on phagocytic ability and bactericidal activity of macrophages by ectopically overexpressing NHE9. Considering the function of NHE9 in serving as a leak pathway for protons, we hypothesized that NHE9 overexpression would impair phagosome acidification and consequently enable bacterial survival within the phagosome. We demonstrate that NHE9 associates with the phagosome early during the maturation process and alkalizes the phagosome lumen to disrupt transport along the microtubules preventing them from reaching the lysosomes. This has direct consequences on the ability of the macrophage to degrade the ingested bacteria. Overall, we present evidence of a novel determinant of phagosomal pH during host–bacteria interaction.

## Results

### NHE9 expression is downregulated upon bacterial infection in macrophages

**Figure 1:**
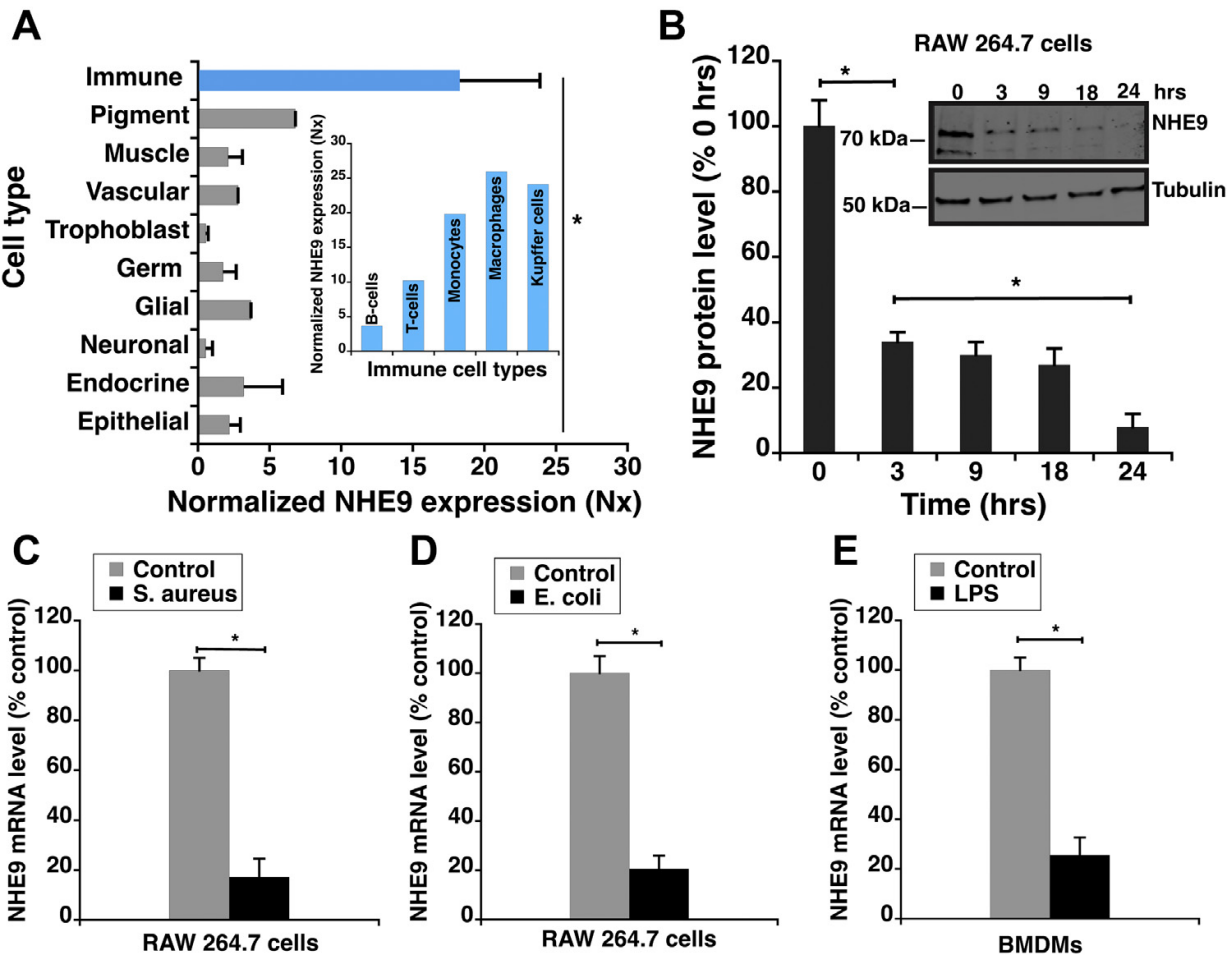
**NHE9 is highly expressed in macrophages but is downregulated upon bacterial infection**. *A*, NHE9 transcript expression profile was obtained from Human Protein Atlas https://www.proteinatlas.org. Normalized expression (Nx) of single cell type transcriptomics data available for various cells within each cell type were averaged. This averaged, normalized expression is shown along with standard deviation accounting for the variance between the data for different cells within each cell type. The inset (*blue*) shows the normalized NHE9 transcript expression for the five types of immune cells available on the Human Protein Atlas, which were averaged to obtain the immune cell type expression (*blue bar*). *B*, NHE9 protein expression levels determined by Western blotting at the indicated times after activation of RAW 264.7 cells with bacterial lipopolysaccharide (LPS), a ligand for the toll-like receptors (TLRs). Graph represents average band intensity from densitometric scans of immunoblots from three biological replicates. Inset shows a representative immunoblot from RAW 264.7 cell lysates showing change in NHE9 expression (*top*) at the indicated times after LPS treatment, with tubulin loading control (*bottom*). Quantitative PCR (qPCR) analysis of NHE9 messenger RNA (mRNA) in RAW 264.7 cells after infection with *S. aureus* (*C*) or *E. coli* (*D*) relative to uninfected control cells. *E*, qPCR analysis of NHE9 mRNA in mouse bone marrow-derived macrophages (BMDMs) after treatment with LPS. Transcripts for qPCR were collected after 24 h postinfection or LPS stimulation. Error bars represent standard deviation (SD). ∗*p* < 0.05. Statistical analysis was done using Student’s *t*-test.

Timely regulation of gene expression is essential for macrophage activation and mounting an immune response (34). Evaluation of single-cell RNA sequencing data, obtained from the Human Protein Atlas for 192 cell type clusters corresponding to different cell type groups https://www.proteinatlas.org (35, 36), revealed that NHE9 transcripts are enhanced in immune cells (Fig. 1*A*), specifically macrophages relative to other cell types (Fig. 1 inset). The high steady-state levels of NHE9 in macrophages are consistent with NHE9’s previously identified roles in endocytic trafficking of plasma membrane receptors and transporters (22, 26, 27, 29). In the context of macrophages, sorting and turnover of pattern recognition receptors, such as the toll-like receptors, is crucial for recognition of microbial pathogens (37, 38). To investigate the role of NHE9 in macrophages, we first analyzed its expression levels after stimulation with toll-like receptor-4 ligand bacterial lipopolysaccharide (LPS), the outer leaflet of the outer membrane of Gram-negative bacteria (39). We observed a sharp decrease in total NHE9 protein levels upon LPS activation of RAW 264.7 cells (mouse macrophage cell line derived from BALB/c mice). Densitometric analysis of Western blot data showed a reduction of NHE9 by more than 60% within 3 h of LPS treatment (Fig. 1*B* and inset). NHE9 protein levels continued to decrease over a 24-h period, with less than 10% of initial NHE9 levels remaining by the 24-h time point (Fig. 1*B*). Next, we tested if our findings are relevant for bacterial infections by monitoring the expression of NHE9 in RAW 264.7 cells after infection with either a uropathogenic strain of *Escherichia coli* (gram-negative bacteria) or *Staphylococcus aureus* (gram-positive bacteria). Consistent with our results from LPS stimulation, we observed a strong decrease in NHE9 transcripts postinfection with gram-positive (Fig. 1*C*) and gram-negative (Fig. 1*D*) bacteria. Finally, we confirmed our findings in primary macrophages. Similar to the trend observed in RAW 264.7 cells, in mouse bone marrow–derived macrophages (BMDMs) upon stimulation with LPS, NHE9 transcript levels decreased significantly (Fig. 1*E*). Overall, we noticed a general trend of NHE9 downregulation in macrophages upon bacterial infection.

### NHE9 expression levels regulate bactericidal activity of macrophages

**Figure 2:**
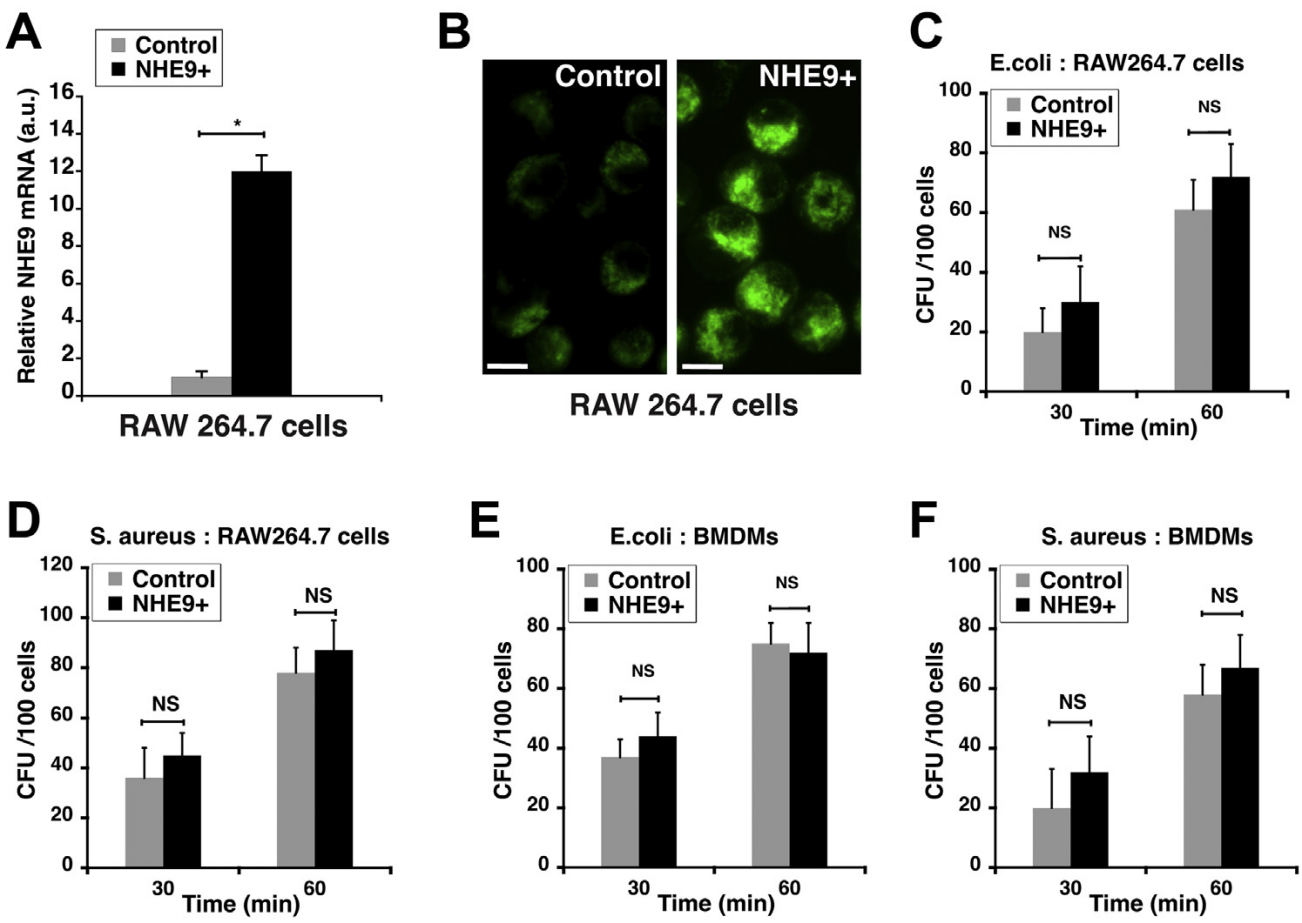
**NHE9 expression levels have no significant effect on pathogen ingestion**. *A*, quantitative PCR analysis of stable, ectopic NHE9 expression in RAW 264.7 cells. Error bars represent standard deviation (SD), ∗*p* < 0.05. *B*, NHE9 expression in RAW 264.7 cells determined by immunofluorescence microscopy. *Left panel*: Control cells showing staining of native NHE9 protein; *Right panel*: Cells stably expressing NHE9 (NHE9+) Scale bar (*white line*) is 10 μm. Phagocytic ingestion by RAW 264.7 cells (*C* and *D*) and mouse bone marrow-derived macrophages, *i.e*., BMDMs (*E* and *F*) after 30- or 60-min incubation with *E. coli* and *S. aureus* bacteria as indicated. Data are expressed as colony forming units (CFUs) per cell. Graphs represent mean from three biological replicates. Error bars represent standard error of the mean (SEM). NS, not statistically significant. Statistical analysis was done using Student’s *t*-test.

**Figure 3:**
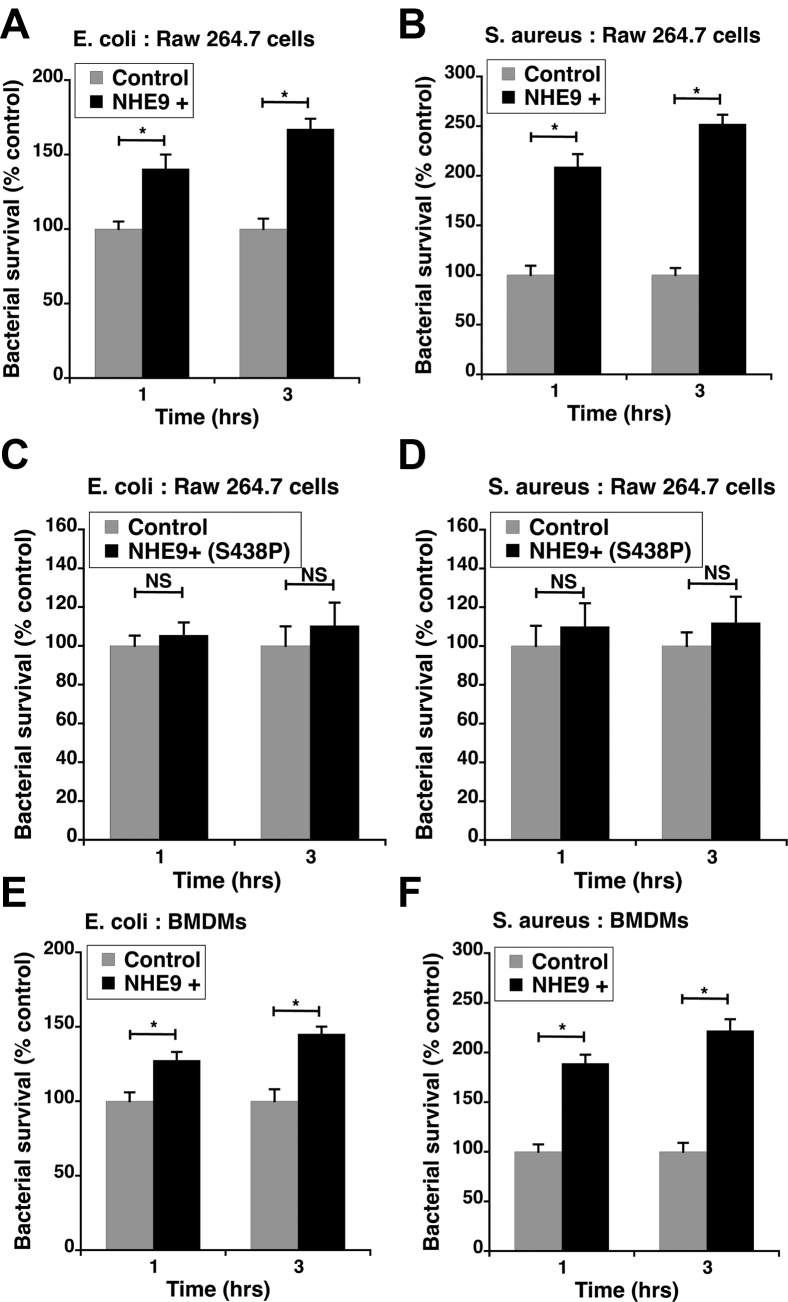
**NHE9 expression regulates bactericidal activity of macrophages**. Surviving *E. coli* (*A*) and *S. aureus* (*B*) 1 h or 3 h postingestion in RAW 264.7 cells, control, and stably expressing NHE9 (NHE9+). (*C*) and (*D*) are the same as (*A*) and (*B*) except the bacterial survival in control cells is compared to cells stably expressing a functional mutant of NHE9 (NHE9+ (S438P)). Surviving *E. coli* (*E*) and *S. aureus* (*F*) 1 h or 3 h postingestion in bone marrow–derived macrophages (BMDMs), control, and ectopically expressing NHE9 (NHE9+). Data are expressed as percent of surviving bacteria, relative to control, determined from colony-forming units as indicated in the methods. Graphs represent mean from three biological replicates. Error bars represent standard deviation (SD). ∗*p* < 0.05. Statistical analysis was done using Student’s *t*-test.

**Fig. S1:**
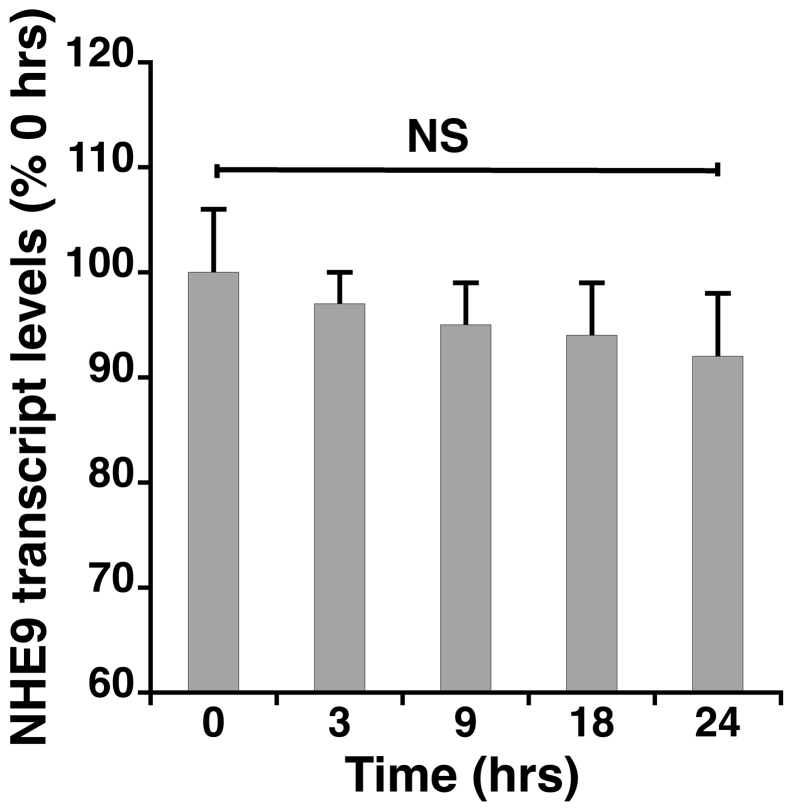


The process of elimination of bacteria within the macrophage can be broadly divided into two phases: (i) ingestion of bacteria leading to formation of nascent phagosomes and (ii) phagosome maturation and subsequent fusion with the lysosome leading to the death of the trapped microbe (1). To determine whether NHE9 expression levels affect either or both phases, we engineered stable overexpression of NHE9 in RAW 264.7 cells, henceforth referred to as NHE9+ cells. Quantitative PCR analysis revealed a 12-fold increase in NHE9 transcripts within NHE9+ cells (Fig. 2*A*). We also confirmed the expression of NHE9 by immunofluorescence (Fig. 2*B*). We confirmed that NHE9 remain highly expressed in NHE9+ cells even after exposure to bacteria (Fig. S1). After, allowing bacterial phagocytosis to occur for 30 min, we assessed the phagosome formation by comparing the presence of live bacteria immediately after ingestion within the control and NHE9+ cells. We did not observe a statistically significant difference in the pathogen burden, for both *E. coli* and *S. aureus*, between control and NHE9+ cells in RAW 264.7 (Fig. 2, *C* and *D*) and BMDM (Fig. 2, *E* and *F*). Next, we compared the bactericidal activity of NHE9+ and control cells by evaluating the pathogen burden 1 h and 3 h after bacterial uptake. The *E. coli* burden was ∼40% (1 h after ingestion) and ∼67% (3 h after ingestion) higher in NHE9+ cells compared to control RAW 264.7 cells (Fig. 3*A*). We noticed a similar trend for *S. aureus* infection of RAW 264.7 cells (Fig. 3*B*). To confirm that the reduced bactericidal activity in NHE9+ cells is a consequence of NHE9’s ion transport function and not a nonspecific effect of overexpression, we evaluated the effect of overexpressing a loss-of-function mutant of NHE9 (S438P) (29). We did not discern a statistically significant difference in bactericidal activity between control and RAW 264.7 cells expressing the functional mutant (Fig. 3, *C* and *D*). These data clearly indicate that an increase in NHE9-mediated ion transport resulted in reduced bactericidal activity in RAW 264.7 macrophages. Next, we confirmed the validity of our findings from RAW 264.7 cells in BMDMs (Fig. 3, *E* and *F*). Our observations from BMDMs were consistent with the findings from RAW 264.7 cells, indicating that NHE9 expression levels affect phagosome maturation but not phagosome formation.

### NHE9 modulates pH of the maturing phagosome

**Figure 4:**
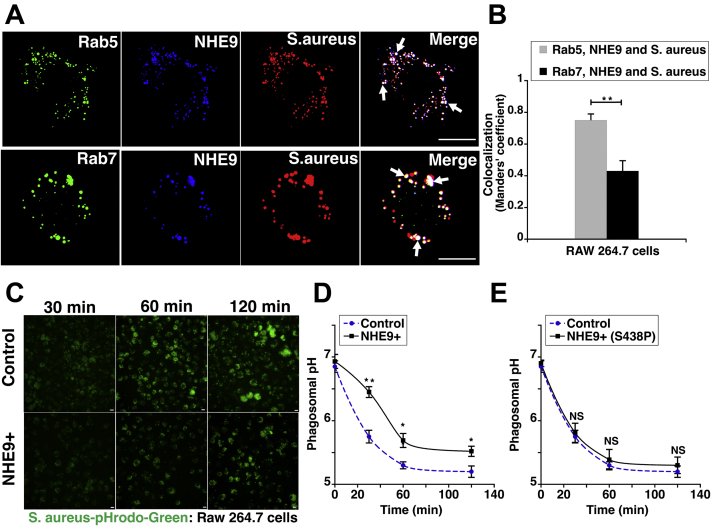
**NHE9 alkalizes the maturing phagosome**. *A*, colocalization in RAW 264.7 cells as determined by immunofluorescence microscopy. *Top panel*: NHE9 (*blue*) colocalizes with BacLight stained *S. aureus* (*red*), and Rab 5 (*green*), a marker for early phagosomes. *Bottom panel*: NHE9 (*blue*) colocalizes with BacLight-stained *S. aureus* (*red*), and Rab7 (*green*), a marker for late phagosomes. Colocalization is indicated by white dots in the merged images (*arrows point* to representative phagosomes). Scale bar (*white line*) is 30 μm. *B*, quantification of colocalization done using Manders’ coefficient. Graph represents mean from three biological replicates, and > 30 cells were used for each replicate. *C*, pH-sensitive fluorescence of pHrodo-green conjugated to *S. aureus* bioparticles after 30, 60, and 120 min in RAW 264.7 cells. *Top panel*: Control cells and *Bottom panel*: Cells stably expressing NHE9 (NHE9+). Panels show representative live-cell images of different cells for the indicated time points. Scale bar (*white line*) is 10 μm. *D*, phagosomal pH was determined from fluorescence of pHrodo-green after calibration with buffers of known pH and normalizing for differences in uptake using *S. aureus* conjugated to pH-insensitive fluorophore (Alexa Fluor 594). Graph shows comparison between phagosomal pH of control *versus* RAW 264.7 cells stably expressing NHE9 (NHE9+). *E*, same as (*D*) except the data shows phagosomal pH of control *versus* RAW 264.7 cells stably expressing NHE9 functional mutant (NHE9+ (S438P)). Graphs represent mean from three biological replicates. Error bars represent standard deviation (SD). ∗*p*< 0.05 and ∗∗*p* < 0.01. Statistical analysis was done using Student’s *t*-test.

To understand the mechanistic basis of how changes in NHE9 expression impact bactericidal activity of macrophages, we first asked, when does NHE9 associate with the phagosome? Previous studies in several primary cell types and immortalized cell lines have shown that NHE9 localizes to both early and late endosomes (23, 26, 27, 28, 29, 40). Nascent phagosomes merge with early endosomes to form the early phagosomes, which sequentially fuse with late endosomes that finally merge with the lysosomes (1, 41). Consistent with the presence on the early phagosome, we observed colocalization of NHE9 with Rab5- (early endosome marker) and BacLight-stained bacteria (Fig. 4*A*-top panel). This trend continued with the late phagosome as evident by NHE9’s colocalization with Rab7 (late endosome marker) in the bacteria hosting compartment (Fig. 4*A*-bottom panel). Quantification of colocalization using the Manders’ overlap coefficient indicated significantly higher localization of NHE9 with the early phagosome relative to the late phagosome (Fig. 4*B*). The phagosome undergoes progressive acidification as it matures (1). Considering the role of NHE9 in transporting protons out of the phagosome lumen, we investigated whether an increase in NHE9 expression results in limiting the acidification of the maturing phagosome. We tracked pH-sensitive fluorescence of pHrodo-green conjugated to *S. aureus* bioparticles for 2 h in RAW 264.7 cells (Fig. 4*C*). Differences in uptake were normalized using *S. aureus* conjugated to pH-insensitive fluorophore (Alexa Fluor 594). Phagosome pH was determined by calibration using buffers of known pH. We found that phagosomes in control cells were consistently more acidic at all the evaluated time points relative to phagosomes in NHE9+ macrophages (Fig. 4*D*). We did not observe a statistically significant difference in pH between control cells and macrophages expressing NHE9 functional mutant (S438P), ruling out any nonspecific effect of overexpression (Fig. 4*E*). These data show NHE9 expression prevents the progressive acidification of the lumenal pH in the maturing phagosome.

### NHE9 impairs maturation-associated transport of phagosomes

**Figure 5:**
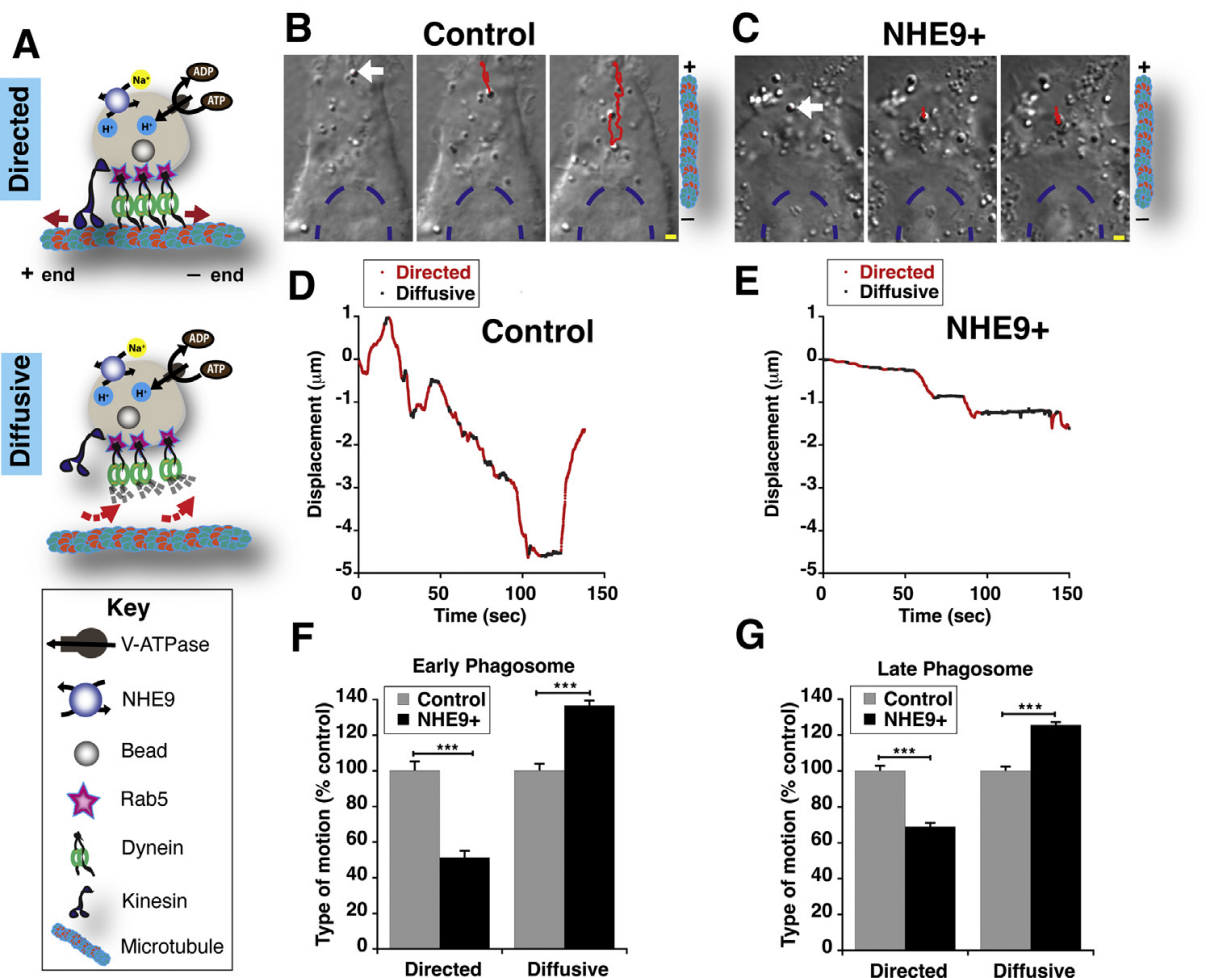
**NHE9 impedes the intracellular long-distance transport of phagosomes.***A*, schematics of directed and diffusive motion of phagosomes observed in cell. *Top panel*: microtubule-based long-distance bidirectional transport of a phagocytosed bead by unidirectional motor proteins kinesin and dynein. Establishment and maintenance of an acidic lumen requires precise co-ordination of H^+^ ion pumping (accomplished by V-ATPase) and countervailing H^+^ leakage by NHE9. *Bottom panel*: diffusive motion of phagosomes due to inactive motor proteins or motor proteins detached from microtubules. *B* and *C*, representative differential interference contrast (DIC) images with overlaid tracked position (*red*) of a centripetally moving, phagocytosed, 810 nm diameter bead (*white arrow*), in RAW 264.7 cells, control (*B*) and stably expressing NHE9 (NHE9+) (*C*). The *dotted blue line* indicates the position of nucleus in each cell. The scale bar (*yellow line*) is 2 μm. (D and E) Displacement-time plots of the tracked positions obtained for the phagocytosed bead from the corresponding cell displayed in panels (*B* and *C*), respectively. The displacement plot for NHE9+ (E) displays reduced directed motion (*red*) and increased diffusive motion (*black*) in comparison to bead motion in control cells (*D*) within the same duration of 150 s. The directed and diffusive motion were determined by using the mean-square displacement approach. *F* and *G*, represent the percentage of directed and diffusive movement of phagosome beads for early (*F*) and late (*G*) phagosomes obtained from both control and NHE9+ cells. Sixty-three early phagosome beads were tracked for both control and NHE9+ cells (number of cells tracked = 23 (control) and 18 (NHE9+)). Over 100 late phagosome beads were tracked for both control and NHE9+ cells (number of cells tracked = 30 (control) and 29 (NHE9+). Five independent experiments for early and eight independent experiments were conducted for late phagosomes. The data were processed using MSD to calculate the number of directed and diffusive segments in each track. Statistical analysis was done using two–sample *t*-test (∗∗∗*p* < 0.001). MSD, mean-square displacement.

Phagosome maturation is mechanistically dependent on successful centripetal motion of the early and late phagosomes to the perinuclear region for fusion with the lysosomes. Lumenal pH of the phagosome is known to signal changes in lipid–protein architecture on the phagosomal membrane, which in turn regulates motor recruitment and transport (42, 43, 44). In this context, we hypothesized NHE9 expression and consequent alkalization would impact phagosome movement on the microtubules. To test this, we monitored the transport of carboxylate-coated polystyrene beads in RAW 264.7 cells. These beads have diameters of ∼800 nm, comparable to the size of bacteria, and can be tracked with subnanometer precision consistently throughout the journey after phagocytosis as they are not degraded inside the cell (45). Once phagocytosed into RAW 264.7 cells, the beads were chased for 5 min or 30 min to investigate early and late phagosome motion, respectively (42). Based on previous reports, we anticipated seeing both directed and diffused motion of phagocytosed beads along the microtubules during the biogenesis of early and late phagosomes (42, 46, 47, 48). Directed motion (Fig. 5*A*, top panel) is caused by microtubule-based molecular motor proteins. Diffusive motion (Fig. 5*A*, center panel) is the result of inactive motor proteins or motor proteins being completely detached from microtubules, leading to random, insignificant net displacement (46, 49, 50, 51). Consistent with previous observations, differential interference contrast imaging in live cells showed a combination of directed and diffused motion of the phagocytosed beads in both control and NHE9+ macrophages (Fig. 5, *B* and *C*). We separated the directed (*red*) and diffusive (*black*) segments in the tracks of phagocytosed beads (Fig. 5, *D* and *E*) using a mean-square displacement approach and calculated the average percentage of directed and diffusive motion for early and late phagosomes (52). Interestingly, within NHE9+ macrophages, directed motion decreased by ∼50% for early phagosomes (Fig. 5*F*) and ∼30% for late phagosomes (Fig. 5*G*), relative to control cells. In contrast, significant increase in diffusive motion was observed in NHE9+ macrophages for both early and late phagosomes (Fig. 5, *F* and *G*).

**Figure 6:**
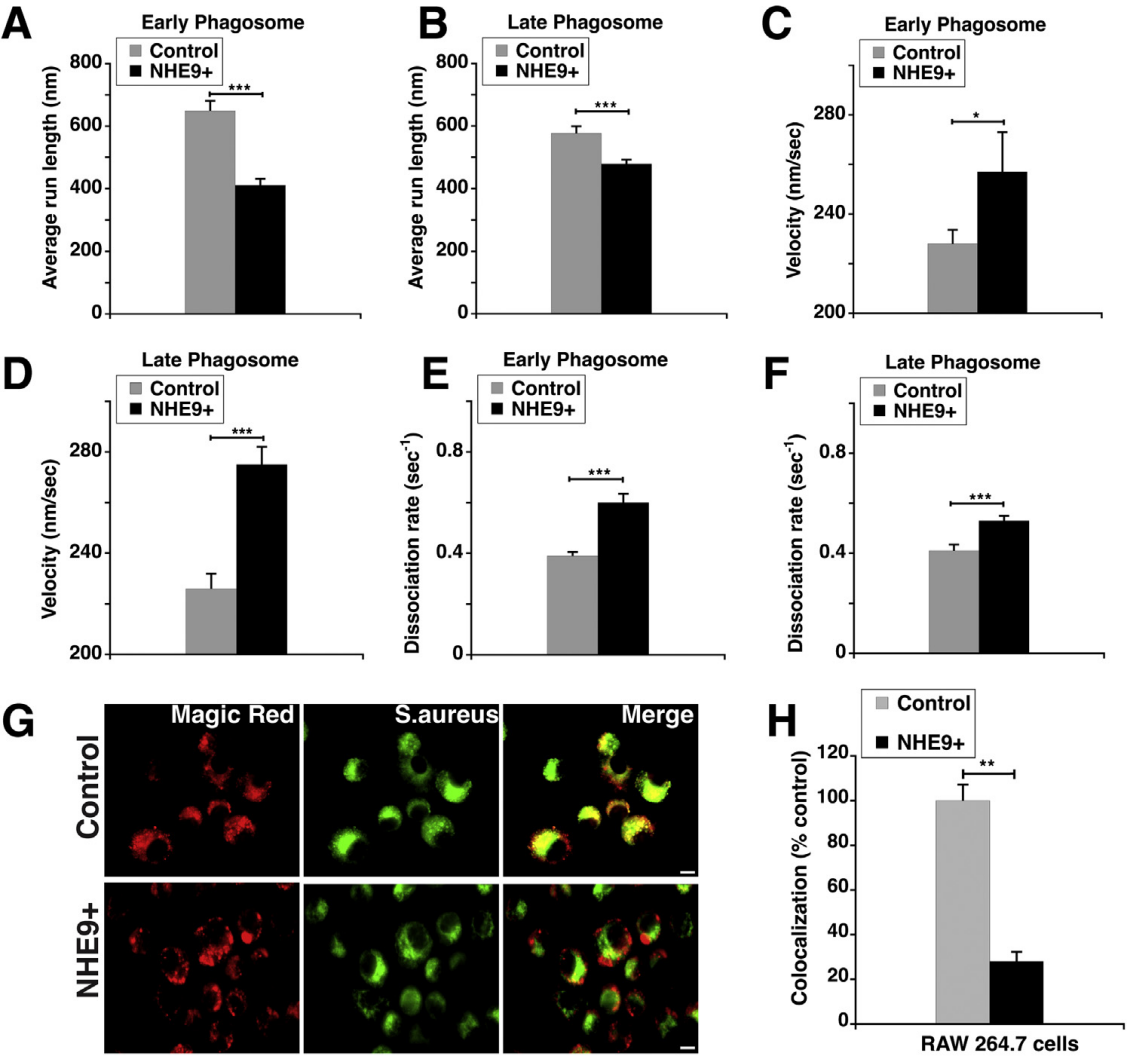
**NHE9 alters the biophysical parameters of phagosome transport**. The biophysical parameters associated with directed movement of phagocytosed beads were characterized where runs greater than 200 nm in *length*, speed greater than 50 nm/sec, and run duration of at least 0.25 s were considered as run segments. Graphs display the comparative run-lengths (*A* and *B*), velocities (*C* and *D*), and dissociation rates (*E* and *F*) in early and late phagosomes as indicated for RAW 264.7 cells stably expressing NHE9 (NHE9+) *versus* control. Data displayed is the mean ± SEM. Directed run segments analyzed for early phagosomes, n = 177 (NHE9+) and 504 (control). Directed run segments analyzed for late phagosomes, n = 922 (NHE9+) and 470 (control). Statistical analysis was done using two–sample *t* test (∗*p* < 0.05 and ∗∗∗*p* < 0.001). Localization of BacLight-stained *S. aureus* (*green*) with Magic *Red reagent* (*red*) in NHE9+ and control RAW 264.7 cells, as determined by immunofluorescence microscopy. Colocalization is indicated by *yellow* in the merged images. Scale bar (*white line*) is 10 μm (*G*). Quantification of colocalization done using Manders’ coefficient (*H*). Graph represents mean from three biological replicates and > 30 cells were used for each replicate. Error bars represent standard deviation (SD). ∗∗*p* < 0.01. Statistical analysis was done using Student’s *t*-test.

Based on the significant loss in directed motion of the phagosomes in NHE9+ cells, we expected these phagosomes to be less successful in reaching the lysosomes. To this end, we first compared the average run lengths of early and late phagosomes in NHE9+ cells relative to control cells. Consistent with a loss in microtubule-based, directed movement, early phagosomes in NHE9+ cells exhibited ∼35% reduced run-length compared to control (393 *versus* 611 nm) (Fig. 6*A*). The trend was similar in late phagosomes: NHE9+ cells had ∼17% reduced run length relative to control (479 *versus* 576 nm) (Fig. 6*B*). Next, we measured the velocities for the run segments of phagocytosed beads. The distribution was quite broad with velocities ranging from 50 nm/sec to 1500 nm/sec for both types of phagosomes. Unlike run lengths, the average velocities in NHE9+ cells were ∼13% and ∼22% higher for early and late phagosomes, respectively (Fig. 6, *C* and *D*). These data are consistent with the possibility of reduction in active motor numbers on the phagosomes contributing to the increased velocities in NHE9+ (42, 53, 54, 55, 56). In multimotor transport systems, the rate of cargo transitioning out of the motor bound state, referred to as the dissociation rate, is reduced by decreasing the single motor velocity (57). Therefore, for phagosomes in NHE9+ cells, we expected that the velocity increases at the expense of motor bound travel on the microtubules. Supporting this, we observed a sharp increase in average dissociation rate for early (∼50%) and late (∼30%) phagosomes in NHE9+ cells relative to control (Fig. 6, *E* and *F*). In sum, these data indicate that high NHE9 expression on phagosomes is associated with loss of active motor proteins and/or detachment of motor proteins from the microtubules. Because of the impaired run on the microtubules, we expected the pathogen transported by the phagosomes in NHE9+ macrophages to be less successful in reaching the lysosomes. To test this, we conducted Magic Red (MR) cathepsin assay in conjunction with fluorescence microscopy (58). The MR reagent is a substrate for cathepsin enzymes in the lysosomes. While the MR substrate itself is not fluorescent, upon cleavage by active cathepsin enzymes in the lysosomes, it fluoresces red. We compared the localization of BacLight stained *S. aureus* with MR in NHE9+ and control cells (Fig. 6*G*). Consistent with phagosomes being less effective in reaching the lysosomes, we observed a ∼72% decrease in localization (Manders’ coefficient) of *S. aureus* to the lysosomes in NHE9+ macrophages relative to control cells (Fig. 6*H*). These results are in accordance with the decreased bactericidal activity we observed in macrophages with high NHE9 expression (Fig. 3, *A*, *B*, *E* and *F*).

## Discussion

Phagosome maturation is crucial to the normal homeostasis of the immune system (59). Acidification is indispensable to phagosome maturation and is not simply a consequence of the process (2). However, the mechanisms that regulate the acidification process and key functions accomplished by phagosomal acidification remain unclear. Identification of specific molecules that regulate phagosomal pH and determining the significance of this regulation in the maturation process within macrophages is central to understanding host–pathogen interactions. In this study, we demonstrate for the first time, a direct role for the monovalent Na^+^(K^+^)/H^+^ antiporter, NHE9, in phagosomal pH regulation and reveal its impact on phagosome transport during maturation.

In immune cells, specifically the macrophages, steady-state levels of NHE9 are significantly high relative to other cell types. Remarkably, upon active infection, we observed that NHE9 expression is strongly downregulated. This is not merely specific to the two pathogens we tested, *E. coli* and *S. aureus*, as previous reports indicate a similar downregulation of NHE9 following *Mycobacterium tuberculosis* and *Brucella abortus* infections (13, 60, 61). Thus, NHE9 downregulation appears to be a general response to microbial ingestion. We demonstrated that a compensatory increase of NHE9 through ectopic expression has direct consequences on the bactericidal activity of macrophages. Mechanistically, we show this is a result of defective trafficking of the phagosome. The data presented establish that lumenal pH in the phagosomes is consistently high in macrophages with elevated NHE9 expression. This correlates negatively with run lengths and directed motion of phagosomes on the microtubules. Furthermore, relatively high velocities and increased dissociation rates were observed in phagosomes expressing NHE9 ectopically. Finally, we show that phagosomes in macrophages with increased NHE9 expression are less likely to reach the lysosomes. Based on these data, we propose that NHE9 exerts control over the number of active motor proteins on phagosomes, thus contributing to the altered motility resulting in fewer productive lysosome encounters.

Though impaired phagosomal transport underlies the noted decrease in microbicidal activity of macrophages with elevated NHE9 expression, disruption in fusogenicity of the phagosome with the lysosome cannot be ruled out. This observation stems from a previous report which showed that changes in expression of the yeast ortholog of NHE9 targets pH-sensitive machinery involved in SNARE-mediated merging of the lipid bilayers on endosomes and lysosomes (62). Therefore, the increased survival we observed could more likely be a result of both impaired transport and fusogenicity of the phagosome. The latter, however, remains to be confirmed. While our study establishes the significance of NHE9-mediated pH regulation in phagosome motility, follow-up studies should probe the identity of the sensor(s) of lumenal pH and dissect the mechanisms through which this information is communicated to effect motor protein recruitment to the phagosome.

Directed motion of the phagosome on the microtubules is bidirectional, with a tug-of-war between dynein and kinesin motor proteins, directing phagosome transport toward and away from the lysosome, respectively (41, 48, 49, 52). Clustering dynein motors in lipid microdomains helps generate large cooperative forces to drive the phagosome toward the minus-end of microtubules for lysosomal fusion (42). Previous studies have shown that luminal acidification negatively correlates with the levels of lipids such as phosphatidylinositol 3-phosphate and cholesterol (44, 63, 64). Such lipids, either directly or through effectors, are involved in recruiting motor proteins (42, 65). It is therefore, tempting to consider that NHE9 protein levels, through pH manipulation, affects phagosome membrane lipid–protein constitution and consequently dynein motor protein recruitment. Testing this hypothesis should be a focus of future studies.

Overall, our work introduces NHE9 as a new regulator of phagosomal pH and draws attention to the impact of lumenal pH on phagosome transport. Manipulation of phagosomal acidification is a strategy used by several unrelated pathogens (1). Moreover, phagosomal H^+^ leakage is a known approach employed by pathogens to subvert lethal acidic encounters in the phagosome (1, 66). Therefore, in the context of host pathogen interactions, improving the resolution of NHE9’s role has important therapeutic implications.

## Experimental procedures

### Cell culture

RAW 264.7 cells (American Type Culture Collection) were cultured in Dulbecco’s modified Eagle’s medium (DMEM, Thermo Fisher Scientific) supplemented with 10% fetal bovine serum (Sigma) and 5% antibiotic–antimycotic (100× stock =10,000 units/ml penicillin, 10,000 mg/ml streptomycin; Gibco). Murine bone marrow–derived macrophages derived from adult C57BL/6 mouse bone marrow (ScienCell Research laboratories) were cultured using Macrophage Medium (ScienCell Research laboratories). The cells were maintained in a 5% CO_2_ incubator at 37 °C. RAW 264.7 cells stably expressing full-length human SLC9A9 (clone from Genecopoeia) were generated by viral transduction, followed by selection with puromycin (5 μg/ml). Murine BMDM were transfected using Lipofectamine LTX transfection reagent (Invitrogen) according to the manufacturer’s instructions. Empty vector was used for control transductions and transfections.

*E. coli* CFT073 was isolated from the blood and urine of a patient with acute pyelonephritis [PMID 2182540] and *S. aureus* strains were used in cell culture experiments. Bacteria were routinely cultured in lysogeny broth medium (per liter; 0.5 g NaCl, 10 g tryptone, 5 g yeast extract). For macrophage infections, bacteria were cultured to mid-logarithmic phase (*A*_600nm_ of 0.5) at 37 °C plus shaking, collected by centrifugation and washed in phosphate buffered saline (PBS) before inoculating macrophages.

### RNA isolation and quantitative PCR

Isolation of mRNA and quantitative PCR analysis were conducted as described previously (26). mRNA was isolated from RAW 264.7 cells and murine bone marrow–derived macrophages using the Rneasy mini kit (Qiagen) following the manufacturer’s instructions with an additional step to remove DNA using Dnase I (Ambion, Thermo Fisher Scientific). cDNA was synthesized using the high-capacity RNA to cDNA kit (Applied Biosystems) following the manufacturer’s instructions. Quantitative real-time PCR analysis experiments were set up using TaqMan fast universal PCR Master Mix (Applied Biosystems) according to the manufacturer’s instructions on CFX connect real time system (Bio-Rad). TaqMan gene expression assay probes used were as follows: Mm00626012_m1 and Hs00543518_m1 (SLC9A9), Mm99999915_g1 and Hs02758991_g1 (GAPDH), and Mm03928990_g1 and Hs03003631_g1 (18S rRNA). Cycle threshold (Ct) values were first normalized to endogenous controls. Fold change was calculated as 2−ΔΔCt, where ΔΔCt is the normalized Ct value relative to control. Three technical replicates of three biological replicates were run to account for variance in assays.

### Immunoblotting

Western blotting was conducted as described previously (26). Briefly, the cells were lysed at the indicated times with mammalian protein extraction reagent (Thermo Fisher Scientific) that included protease inhibitor mixture (halt protease inhibitor mixture, Thermo Fisher Scientific). Lysates were centrifuged at 15,000 r.p.m. for 10 min (4 °C). Cell protein lysates (∼100 μg) were dissolved in loading buffer (62.5 mM Tris-HCl, pH 6.8, 10% glycerol, 2% SDS, 0.01% bromophenol blue, 100 mM DTT) and separated by SDS-polyacrylamide gel electrophoresis. Antibodies used were SLC9A9 Polyclonal Antibody (PA5-42524, Thermo Fisher Scientific) at 1:50 dilution and monoclonal α-tubulin antibody (T 9026, Sigma) at 1:500 dilution.

### Immunofluorescence and microscopy

RAW 264.7 cells on coverslips were washed with PBS and fixed for 15 min at room temperature with solution containing 4% paraformaldehyde. Permeabilization was conducted after cold PBS washes with 0.1% Triton-X in PBS for 5 min. Next, the cells were incubated for 30 min in block solution (1% BSA, 0.3 M glycine, and 0.1% Tween 20). Following this, cells were incubated overnight at 4 °C with NHE9 antibody at 1:100 in block solution (PA5-42524, Thermo Fisher Scientific). Following PBS washes, Alexa Fluor-conjugated secondary antibodies (Invitrogen) were used at 1:1000 dilutions for 30 min. The cells were mounted onto slides using VECTASHIELD Antifade Mounting Medium (Vector Labs). For colocalization studies, *S. aureus* were stained with BacLight Red Bacterial Stain (Thermo Fisher Scientific) according to the manufacturer’s instructions. Stained bacteria were incubated with activated RAW 264.7 cells at a 1:1 ratio in serum-free culture media for 45 min. RAW 264.7 cells were then washed with 12.5 μg/ml gentamicin (39) to remove unbound bacteria and then incubated for different durations as indicated, prior to fixing and incubation with primary antibodies for NHE9, Rab5 (Thermo Fisher Scientific), and Rab7 (Sigma) as described above. Images were collected using a fluorescence microscope (ECHO Revolve). For lysosomal staining, live cell imaging (Lumascope 620, Etluma) was conducted on RAW 264.7 cells dually stained with BacLight Green Bacterial stain (treated as described above) and Magic Red substrate (Bio-Rad). MR treatment was conducted for 1 h, following manufacturer’s instructions. Co-localization was analyzed using ImageJ software (67) by randomly scanning >30 cells in each test group in at least three independent experiments.

### Phagocytosis and bactericidal activity assays

The efficiency of phagocytosis and intracellular bacterial killing was analyzed by counting of bacterial colonies as described previously (39, 68).

Phagocytosis (bacterial ingestion) assay: Mid-log phase bacterial cultures were incubated with activated macrophages cultured in 12-well plates at an multiplicity of infection of 10 for 30 or 60 min as indicated. After the indicated time, supernatant was aspirated, wells were washed 3× with PBS to remove extracellular bacteria, and media containing gentamycin (12.5 μg/ml) was added for 30 min to kill any remaining extracellular bacteria. Next, the cells were washed with PBS and lysed with Triton-X. The numbers of phagocytosed live bacteria were determined by colony count assay. Cell lysates in a series of 10-fold dilutions were plated in triplicate on agar plates followed by incubating overnight at 37 °C. The phagocytosis efficiencies were identified as bacterial colony forming unit per cell at 30 min and 60 min postinfection.

Bacterial killing assay: To determine the efficacy of intracellular killing of bacteria, after removal of extracellular bacteria as described above in the phagocytosis assay, cells were thereafter incubated for another 1 h or 3 h as indicated in complete cell culture media, and then lysed to plate overnight at 37 °C. The surviving bacteria were calculated as colony forming unit per cell.

### Phagosome pH measurement

Phagosomal pH was determined using pHrodo-green–conjugated *S. aureus* bioparticles (Thermo Fisher Scientific) following the manufacturer’s protocol for fluorescence imaging. Bioparticles were added at a concentration of 100 μg/ml in cell culture media. Activated RAW 264.7 cells were pulsed with the bioparticles for 30 min then extensively washed in cold PBS. The cells were then chased for 30, 60, and 120 min as indicated. To normalize for total bioparticle uptake, *S. aureus* conjugated to pH-insensitive Alexa Fluor 594 (Thermo Fisher Scientific) was pulse chased as described above for pHrodo-green conjugated *S. aureus* bioparticles. Live Cell Imaging Solution (Thermo Fisher Scientific) with 20 mM glucose and 1% BSA was used to rinse the cells, following which fluorescence images were acquired with Lumascope 620 (Etaluma). Internal fluorescence was quantified using ImageJ software (67), and average fluorescence intensity was recorded. A pH calibration buffer kit (Thermo Fisher Scientific) was used to generate a standard curve from which phagosomal pH was estimated.

### Motility of bead-phagosomes

#### Sample preparation and live-cell imaging

RAW 264.7 cells were plated on Poly-L-Lysine (Sigma) coated coverslips (22 × 30 × 0.17 mm) in culture dishes and used for phagosome preparation at 50% confluence. Carboxylate polystyrene beads of 810 nm diameter were washed with serum-free DMEM media and incubated with activated RAW 264.7 cells at 1:1000 dilution for 30 min. Next, the cells were washed with PBS, and the incomplete DMEM was then exchanged with complete culture media. Following this, a chase of 5 min and 30 min was given for early and late phagosomes, respectively (39, 44). The pulse and chase were done at 37 °C in the cell culture incubator. The dishes were then washed thrice with ice-cold PBS. The coverslip with the cells from the culture dish was placed in a chamber on a glass slide, with cells facing the inside so they stayed in the medium. The chamber was prepared using double-coated adhesive tape (3M10587-ND, Digi-Key, 25 μm thickness) on a microscope glass slide (1 × 25 × 75 mm), and about 50 μl of warm cell culture media was added to it (55). The sides of the chamber were sealed by vacuum grease. The sample was placed on a Nikon TE300 inverted microscope equipped with a three-axes piezo-electric stage (BIO3, PIEZOCONCEPT) preheated to 37 °C using a custom-made heater plate which accommodated a 50 mm diameter ring heater plate (HT19R2, Thorlabs). The cells were imaged using 1.3 NA 100× objective for about an hour. Differential interference contrast images of the phagocytosed beads were recorded at 20 frames per second using an NIR camera with a spatial resolution of 27.6 nm/pixel. Each cell was recorded for 20 min. The microscope system was settled on an optical table (T46HK, Thorlabs,) which was floated on active-isolating optical table supports (PTS601, Thorlabs) to isolate the sample from all external vibration sources.

### Characterization of phagosome movements

The position signal of phagocytosed beads in cells were obtained with sub-nanometer precision by tracking their movements using the centroid position method (69). The obtained position tracks (Fig. 5, *D* and *E*) reveal the presence of directed as well as diffused motion (52). The diffused and directed segments were separated using the mean-square displacement (MSD) approach (52, 70). Using a custom-built LabVIEW code, the position tracks were scanned with a sliding window of 2.0 s (40 frames) to capture the heterogeneity of bead movements. For each sliding window, the calculated MSD *versus* time plot was fitted with the equation

(1) $$MSD=\;\left(4D\right)t^{\alpha }+2\;\sigma^{2}$$

where α is the scaling exponent, D is the diffusion constant, and σ is the error in the measurement which was fixed to 9.8 nm (Fig. S2*A*). The viscoelastic nature of the cytoplasm leads to a wide range of scaling exponents (Fig. S2*B*) (50, 71). The distribution of scaling exponents showed major peaks at α∼1 (pure diffusion) and at α∼2 (directed motion). To separate the directed from diffusive motion, the distribution of α was fitted with the sum of two Gaussian functions, and the cutoff values (α=1.41 for early phagosomes, and α=1.53 for late phagosomes) were determined. MSDs of separated segments (n=1500 for both diffusive and directed motion) from randomly selected position tracks were averaged. The averaged MSDs were fit by (Equation 1), and the average scaling exponent components α=1.15 for diffusion and α=1.84 for directed motion were determined (Fig. S2*C*) to show the validity of the procedure used.

To further extract the biophysical parameters from the separated directed segments, consecutive segments were combined to build long directed position tracks that were further parsed into segments of constant velocity using the Bayesian optimization technique (72, 73). The criteria used for a displacement to be scored as a run were that the directed movements needed to last for at least 0.25 s, and the velocity had to be at least 50 nm/s (74). Run segments with velocity smaller than 50 nm/s were considered as pause segments. The microtubule orientation was not confirmed in our analysis, and hence, no separation of tracks based on microtubule minus end (toward perinuclear region) or plus end (away from perinuclear region) motion was performed. All data shown in main graphs are the mean ± SEM. Statistical significance was determined using a two-sample *t* test [not significant (ns), ∗*p* < 0.05, ∗∗*p* < 0.01, ∗∗∗*p* < 0.001, and ∗∗∗∗*p* < 0.0001].

## Data availability

The authors confirm that the data supporting the findings of this study are available within the article and its supporting information.

## Supporting information

This article contains supporting information.

## Conflict of interest

The authors declare that they have no conflicts of interest with the contents of the article.

## Figures and Tables

**Fig. S2A:**
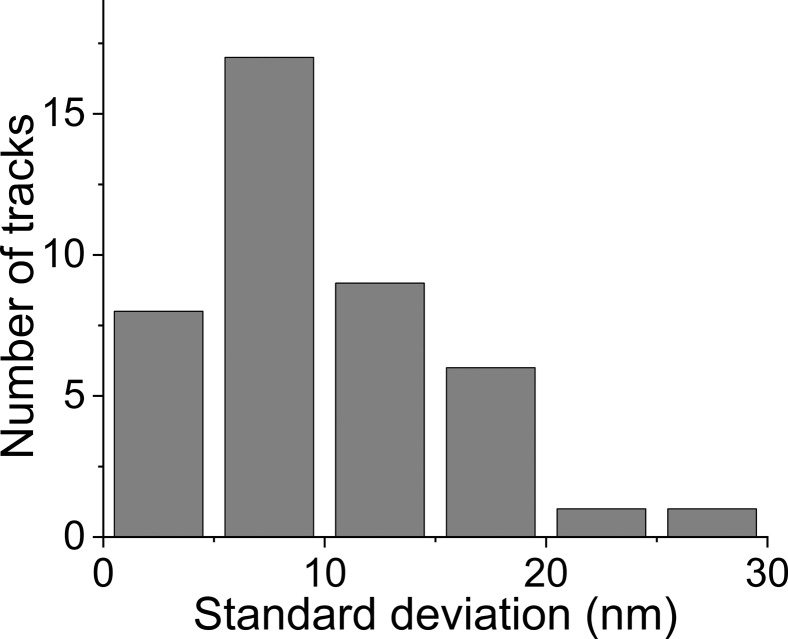


**Fig. S2B:**
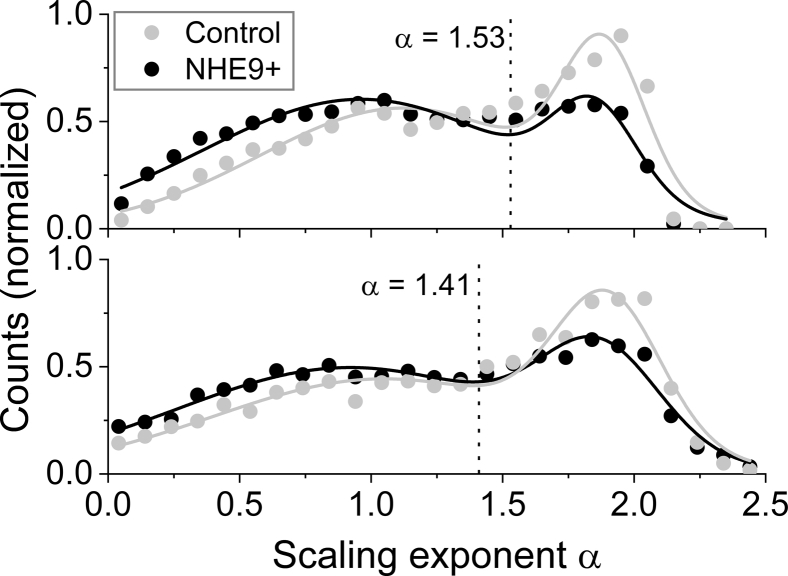


**Fig. S2C:**