# Supplementary material for: The sodium proton exchanger NHE9 regulates phagosome maturation and bactericidal activity in macrophages
Source: J Biol Chem. 2022 Jun 16;298(8):102150. doi: 10.1016/j.jbc.2022.102150 (PMC9293770; doi:10.1016/j.jbc.2022.102150)
Supplement: Supporting Information [file mmc1.docx]

**Supporting Information**

## Figure Legends

**Fig. S1.** Quantitative PCR (qPCR) analysis of NHE9 messenger RNA (mRNA) in RAW 264.7 cells stably expressing NHE9 (NHE9+ cells) after infection with *S. aureus.* Transcripts for qPCR were collected after at the indicated times post infection. Error bars represent standard deviation (SD). NS=Not significant. Statistical analysis was done using Student’s t-test.

##### Method of characterizing movements of phagosome beads

**Fig. S2A.** The measurement error was determined by calculating the standard deviation of 10 second duration position signals obtained from 810nm diameter beads immobilized on to coverslips. All experimental parameters were kept the same as the actual experiment conducted for immobilized live cells (10 beads from 3 independent experiments)

**Fig. S2B.** Distributions of scaling factor 𝛼 for early (lower) and late (upper) phagosomes. The solid lines are the sum of two Gaussian fits. The dashed line divides the regions of directed and diffusive runs with cutoff values $\alpha=1.41$ for early and $\alpha=1.5$3 for late phagosome position trajectories.

**Fig. S2C.** Mean-square displacement (MSD) graph of directed and diffusive segments (n=1500) of 2.0 s period obtained from phagocytosed bead trajectories. The symbols (solid dot for directed motion and triangle for diffusive motion) represent the experimentally measured values (mean ± SEM) and the solid lines are fits using MSD equation.
